# The Whole Heaven a Musical Scale and a Number

**DOI:** 10.3201/eid1605.AC1605

**Published:** 2010-05

**Authors:** Polyxeni Potter

**Affiliations:** Centers for Disease Control and Prevention, Atlanta, Georgia, USA

**Keywords:** Art science connection, emerging infectious diseases, art and medicine, Judith Leyster, Boy Playing the Flute, Dutch Golden Age, respiratory infections, childhood diseases, music of the spheres, about the cover

**Figure:**
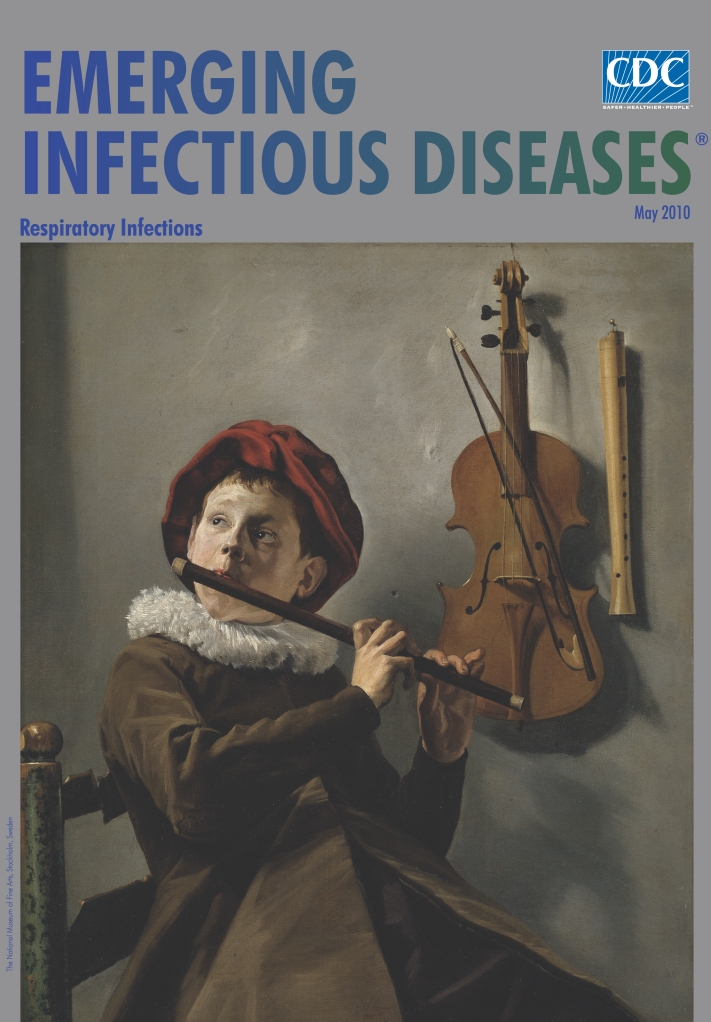
**Judith Leyster (1609–1660) Boy Playing the Flute (1630–1635)** Oil on canvas (73 cm × 62 cm) The National Museum of Fine Arts, Stockholm, Sweden

A woman ahead of her times, Judith Leyster was born the last child, a prodigy, in the large brood of a cloth maker and brewer in Haarlem. While still in her teens, she earned favorable mention in Description and Praise of the City Haarlem in Poetry, a book by Dutch poet Samuel Ampzing. She received art training, probably from Frans de Grebber and may have worked in the studio of well-known portrait painter Frans Hals. In her early 20s, she was admitted into the Haarlem painters’ Guild of St. Luke, soon had her own studio and apprentices, and earned a living as master painter. Her work―mostly portraits and genre scenes filled with merriment―was influenced by the Utrecht school, the followers of Caravaggio, who adopted the Italian master’s use of chiaroscuro. Despite her accomplishments and a measure of fame during her lifetime, Leyster was forgotten after her death at age 50, many of her works mistakenly attributed to other artists. She was rediscovered toward the end of the 19th century. Few of her paintings have survived.

Leyster was innovative, not only in her range of subjects, which was broader than that of her contemporaries, but also in her interpretation of genre. Beyond the usual tavern scenes popular with the buying public, she favored women at home, not a usual theme in Holland until the 1650s. Her view of Dutch domesticity was informal and engaging, subtle and intimate; her use of light and shadow dramatic. Her subjects displayed emotion, liveliness, and a confidence and flair that extended to the distinctive monogram of her signature, the letter “J” capped with a star (lode star or lead star)―a playful reference to her last name.

Genre scenes provide a thorough look into all aspects of the 17th century; music for one, which was ubiquitous in both life and art. Since no recordings were available, music was often spontaneous and always live in homes, taverns, or the outdoors. Scenes containing dance or other amusements always featured musicians and their instruments. Music was woven into the fabric of society and lent itself comfortably to teaching moral ideals so vital to the prevailing Calvinism: “As the old have sung so pipe the young.” Musical instruments were common too in artists’ studios. Painters struggling to raise the social rank of their profession above craft used them to show their understanding of music and solidify their connection with the liberal arts.

Music’s power to inspire and animate originated in antiquity with the writings of Pythagoras, who discovered the physical relationship, expressible in ratios, between mass and sound. “[The Pythagoreans] saw that the … ratios of musical scales were expressible in numbers [and that] … all things seemed to be modeled on numbers, and numbers seemed to be the first things in the whole of nature, [they] supposed the elements of number to be the elements of all things, and the whole heaven to be a musical scale and a number.” Accordingly, the distances between planets would have the same ratios as produced harmonious sounds in a plucked string. The stars and planets, rotating en masse, would produce cosmic harmonies, termed music of the spheres, not accessible to the human ear.

Artists, perhaps intrigued by this celestial connection, have often painted musicians with their eyes lifted upward as a sign of spirituality. Instruments were often used as symbols: strings associated with Pythagorean harmony, wind instruments the province of shepherds and peasants. One of the most musical of her artistic contemporaries, Leyster showed her love of instruments and their sounds in many of her works. She painted them with virtuosity within the recurring theme of music, though she took liberties with the usual classification, marrying such antipodes as the violin and recorder or linking inspiration with the flute.

In Boy Playing the Flute, on this months’ cover, she expertly painted several instruments, along with the performance of a young musician. Balance and harmony in the scene are achieved not by the single figure alone playing the transverse flute but by an ensemble, two of the instruments hanging prominently on the wall beside him, a violin and a recorder. The violin, with its elongated sound holes and exaggerated points, bow threaded behind the strings and no chin rest, seems a fine period specimen, as does the recorder, with its strikingly long windway and smoothly convex foot. The recorder was signed, according to convention, near the mouthpiece by its maker, Leyster. The instrument held reverently by the boy is a Renaissance flute cross-blown and stretched diagonally across the painting. Hues of carob and honey in the clothing and instruments radiate warmth on to the pock-marked wall.

The boy sits awkwardly gazing toward the light. Like all children of this period, he is dressed with the sobriety and modesty of a small adult in velvet coat, linen collar, and a deep red hat, the only relief in the drab attire. His sleeve has been discreetly darned at the elbow, and the chair has seen better days, its painted post discolored and back slat broken, causing him to slouch. Declining affluence aside, this boy is in comfortable enough circumstances. He seems focused on the task, rapt even, as he pushes air through the mouth and manipulates it with slender sensitive fingers to make music. His face, framed between the red hat and white ruff collar is a beacon. His earnest expression belies the wind instrument’s lowly associations.

During this its golden age, Holland’s children were not much better off than most of their other European counterparts, even though progress was made in some areas that affected their survival. The age saw a rebirth of medicine, and for the first time since Hippocrates, clinical observations became important. Influenza, chorea, scarlet fever, scrofula, and pertussis were recognized diagnoses, even though we now know they are not all specific diseases. Franciscus Sylvius, Dutch physician and scientist, identified characteristic changes in the lungs caused by consumption, rampant at the time.

Yet childhood was hardly celebrated as a stage of life, marred as it often was by poverty, social inequity, and high death rates, especially from infectious diseases. Children were viewed as unfinished creatures to be raised into adults. Parenthood was a public virtue, and a basic education, including private music-making for devotional or recreational purposes, was thought good for moral development. Nonetheless, by age 10, most boys were transferred to a master to learn a trade, and poor or orphaned children had already entered the labor force.

Leyster and some of her contemporaries portrayed children sympathetically, allowing them expressions and emotions, music and art, at a time when many of them did not survive their early years. The child in Boy Playing the Flute has fallen on hard times. He is teetering between poverty and comfortable domesticity. Though he still has a home with a violin hanging on the wall, there is no music master in the room. He is going it alone. But “You sing what you hear,” and he hears the harmony above.

While this young musician navigates changing fortunes, he still has an advantage over many of his contemporaries. He is healthy, having so far escaped the scourges of his times, though impending poverty undoubtedly puts him at risk. Pneumonia, diphtheria, diarrheal diseases, tuberculosis, and streptococcal infections were major causes of childhood illness and death, as they still are in much of the world. Some, among them respiratory infections that might have cut the youth’s flute career short, are now under control because of expansive vaccination programs, including in today’s Netherlands.

But while a young boy might tab into the music of the spheres for inspiration and survival, those who try to break the axiomatic connection between wealth, poverty, health, and illness can only rely on programs aimed at the great killers of children. Persistent and unrelenting disharmony, caused by diseases upon diseases emerging and reemerging amidst endless complicating factors, interferes in public health with their orderly conquest, one by one, in any kind of anticipated pattern or ratio on a cosmic scale.
